# The British Journals: Anatomy and Physiology

**Published:** 1839-07

**Authors:** 


### III. THE BRITISH JOURNALS.

(FOR THE QUARTER ENDING MAY 31, 1839.)

### ANATOMY AND PHYSIOLOGY.

### *Experimental Investigation into the Functions of the Eighth Pair of Nerves.* By John Reid, M.D.

This series of experiments was performed in continuation of those formerly published by the author, of which we inserted an abstract in our Fifth Volume (p. 308). A brief account of them was given by Dr. R. at the Newcastle Meeting of the British Association, where they excited considerable attention. They are of so interesting and important a character that we shall present their results somewhat in full; referring, as before, to the original memoir for the evidence by which these are supported. In saying that the excellence of this paper fully bears out the opinion which we formerly expressed as to the merits of the author as an experimental physiologist, we do not perceive that we can offer higher commendation. Dr. Reid possesses, in a remarkable degree, the qualities required for his task—ingenuity in devising experiments, skill in executing them, and caution in reasoning upon them; a combination which is wanting in too many of those who are held up as luminaries of physiological science. We are satisfied that any one who will compare, without prejudice, the two unpretending papers of Dr. Reid with the boasted and boastful *Leçons* of M. Magendie, will not fail to draw conclusions in regard to each of these points by no means favorable to the latter.

The experiments related in this paper almost entirely concern the functions of the pulmonary and gastric branches of the par vagum—those *quæstiones vexatæ* of neurology. A few additions are made, however, on the subjects of the former series; to these we shall first advert.

*Glosso-pharyngeal Nerve.* Dr. Reid has now frequently succeeded in producing *the usual muscular movements* of deglutition, by pinching the trunk of this nerve. He had before only produced convulsive twitches. As he has fully proved that it is not a motor nerve, but acts on the muscles by reflexion, it may evidently be regarded as the principal excitor of the movements of deglutition. That irritation of the trunk of the nerve should have less power of exciting them than impressions made on its extremities, harmonizes well with what we learn from other sources of the character of this class of actions.

*Pneumogastric Nerve.* With regard to the general character of this nerve, Dr. R.'s subsequent experiments have fully confirmed his former opinion, that it ministers to sensation; indications of severe suffering being given when its trunk is pinched, cut, or stretched. He finds an explanation of the negative results which some experimenters have obtained, in the fact that some animals appear much less sensitive to pain than others, even of the same species. "I have experimented on dogs which have endured the incisions necessary to expose the sheath of the carotid artery, without any apparent uneasiness." Magendie has made similar observations: "Such facts ought to make us hesitate before arriving at negative conclusions upon the sensibility of nerves, and forcibly point out a serious error to which limited observations are liable."

*Laryngeal branches.* On the functions of these, also, Dr. R. has obtained additional proof of the correctness of his views. He has found that, after the first paroxysm occasioned by division of these nerves, a period of quiescence and freedom from dyspnœa often supervenes, the respirations being performed with ease as long as the animal remains at rest; but an unusual inspiratory movement, such as takes place at the commencement of a struggle, induces immediate symptoms of suffocation, the current of air carrying inwards the arytenoid cartilages, rendered passive by the paralysed state of their muscles, so as to obstruct the opening of the glottis.

*Cardiac branches.* From seven experiments upon dogs, the following inference is deduced, which is opposed to that of M. Brachet: "After section of the vagi the pulsations of the heart may not only be quickened by muscular exertion, but also by mental emotions. Though in all probability the vagi are the usual channels through which mental emotions affect the heart, yet it appears that this may also take place through the medium of the ganglionic system of nerves."

*Pulmonary branches.* The author confirms his former statement that lesion of one of the pneumogastrics does not necessarily or even generally induce disease of the lung on that side; and he then discusses at much length the effects of lesion of this nerve upon the *respiratory movements.* A large number of experiments were performed to determine this point; and it was an object in these to avoid the disturbance arising from the paralysis of the laryngeal muscles as much as possible. The details of Dr. R.'s method of operating are given by him, that those who wish to test the value of the facts ascertained by him may repeat his experiments in a similar manner. "It is not so much," he remarks, "the frequency of false observations that we have to complain of in medical science as the errors which arise from making these observations under dissimilar circumstances, and from drawing general conclusions from insufficient data."

The first and most positive effect of division of the pneumogastric nerves upon the respiratory movements is a decided *diminution in their frequency.* The number of inspirations soon after the operation was, in the majority of the experiments, reduced about one half. In some cases the number of respirations was afterwards still further reduced, while in others it varied at different periods; and in a few it became more frequent shortly before death. The movements themselves are performed more slowly, in a prolonged and heaving manner, so as to give rise to the idea entertained by some experimenters, and by Dr. R. himself in the first instance, that they are more numerous. It is sufficiently evident, from these experiments, that the vagi are important nerves in transmitting those impressions to the medulla oblongata which excite the respiratory muscular movements. Dr. R. then goes on to prove, by experiments, that Dr. Cruveilhier and Dr. M. Hall are incorrect in asserting that respiration will not go on if the cerebrum and cerebellum are destroyed and the pneumogastrics divided; a contrary result having been obtained by him. The removal of the encephalon diminishes the frequency of these movements, however, whether before or after the section of the vagi. In kittens of a day old they were at first about 100 per minute; after the destruction of the brain and cerebellum they fell to 40; and on cutting the pneumogastrics they instantly fell to between 3 and 4 in the minute, and continued so for some time. It is thus proved that, although the pneumogastric nerves are the *principal,* they are not the *sole* excitors of the respiratory movements; and that there are others which act through a channel in which the encephalon is not concerned. In one of the experiments the spinal cord was divided high up in the neck after the removal of the brain and the division of the pneumogastrics. Respiratory movements were still performed, though with diminished frequency. This fact seems to confirm the doctrine of Dr. M. Hall, relative to the function of the cutaneous nerves, especially those of the face, as excitors of respiration. It is not certain, however, how much of the effect is to be attributed to the sympathetic, which, as has been recently proved by microscopic examination, contains many filaments of the cerebro-spinal system. The diminution in the frequency of the respiratory movements subsequent to division of the vagi has been noticed by other observers; but its constancy has not previously been shown, nor has it been pointed out that it *instantly* supervenes (unless the animal be excited by struggling), and thus cannot result from the circulation of dark blood through the nervous centres.

The next question discussed is that of the *motor* character of the pulmonary branches of the vagus. Regarding this Dr. Reid has not been able to obtain any positive evidence on either side. Certain pathological phenomena would lead to the conclusion that these nerves are capable of exciting muscular contraction in the bronchial tubes; but experiment does not bear out this supposition, though it does not forbid it. The affirmative results obtained by M. Brachet are not regarded by Dr. R. as worthy of confidence. With regard to the *sensory* functions of these branches, Dr. R. is of opinion that, although they are concerned in producing the sensation of "le besoin de respirer," this is *not* annihilated after their section, since animals will even then exhibit great uneasiness if the access of air to their lungs be prevented. The fallacy of the contrary results obtained by Brachet and Mr. Grainger is pointed out. We would suggest that this uneasiness may be produced by impressions transmitted from other parts of the system as well as the lungs, when imperfectly arterialized blood is circulating through the systemic capillaries, just as the sense of hunger seems to depend not only upon the emptiness of the stomach, but upon the demand for nutrition existing in the system at large. The sensibility of the mucous membrane of the trachea and bronchi appears to be impaired by section of the vagi; but it is always much inferior to that of the larynx; and the experiments made to test it are liable to a remarkable fallacy, arising from the fact that the air-passages are easily brought to tolerate stimulants which at first excite great uneasiness.

*Morbid Changes in the Lungs* consequent upon section of the pneumogastrics. The real character of these and the mode in which they are induced constitute a very interesting subject of investigation; and one whose importance in pathology is at once apparent. We are inclined to place the more reliance upon Dr. Reid's statements, as his office as Pathological Clerk at the Royal Infirmary of Edinburgh gives him peculiar opportunities of becoming conversant with morbid appearances, and induces accuracy in the statement of them. In fifteen out of seventeen animals experimented on, the lungs were found more or less unfit for the healthy performance of their functions. Of the two animals in which the lungs were found healthy, one died after the completion of the eighth day, apparently from inanition; the other was killed after the twelfth day, when it was apparently in perfect health. "The most common morbid changes found in the lungs of these animals was a congested state of the blood-vessels, and the effusion of frothy serum into the air-cells and bronchial tubes. In eight out of the fifteen these changes were strongly marked. In some portions of the lungs the quantity of blood was so great as to render them dense. The degree of congestion varied in different parts of the same lung, but was generally greatest at the most depending portions. The condensation was generally greater than what could be accounted for by the mere congestion of blood in the vessels, and probably arose from the escape of the solid parts of the blood into the tissue of the lung." In some instances the condensation was so great that a considerable portion of the lungs sunk in water and did not crepitate; but they did not present the granulated appearance of the second stage of ordinary pneumonia. In five cases in which the animals had survived a considerable time, portions of the lungs exhibited the second and even the third stages of pneumonia, with puriform effusion in the smaller bronchial tubes; and in two gangrene had supervened. "One of the most important points to ascertain in an investigation of this kind is the first departure from the healthy state; to decide whether the effusion of frothy reddish serum, by interfering with the usual changes of blood in the lungs, *causes* the congested state of the pulmonary blood-vessels and the laboured respiration; or whether this effusion is the *effect* of a previously congested state of the blood-vessels." In several of the experiments but a very small quantity of frothy serum was found in the air-tubes, even when the lungs were found loaded with blood, and when the respiration before death was very laboured. "This naturally leads us to doubt whether the frothy serum is the cause of the laboured respiration and the congested state of the pulmonary vessels in those cases where it is present, though there can be no doubt that, when once it is effused, it must powerfully tend to increase the difficulty of the respiration, and the impeded circulation through the lungs." Dr. R. has satisfied himself of an important point which has been overlooked by others, "that this frothy fluid is not mucus, though it is occasionally mixed with it, but is the frothy serum so frequently found in cases where the circulation through the lungs has been impeded for some time before death. From this and other facts Dr. R. concludes "that the congestion of the blood-vessels is the first departure from the healthy state of the lung, and that the effusion of frothy serum is a subsequent effect." What then is the cause of this congestion and of the retardation of the movement of the blood? We fully agree with Dr. R. in considering the diminished frequency of the respiratory movements, and the consequent check to the aërating changes as sufficient to account for it, since "it is an established fact that the flow of the blood through the lungs is dependent upon the continuance of the respiratory process." It has been supposed by some to be due to paralysis of the muscular fibres of the bronchial tubes; after every endeavour to obtain distinct evidence of this, however, he has been unsuccessful, though he does not deny the possibility of such an influence. Several cases of disease in man are mentioned by him, in which the lungs presented after death a condition similar to that observed in the lower animals after section of the vagi; and in these the respiratory movements had been much less frequent than natural during the latter part of life, owing to a torpid condition of the nervous centres. He controverts the opinion of Wilson Philip that the effusion is the result of a morbid secretion immediately consequent upon interruption of nervous influence, and points out how galvanism may operate in preventing it. The true mucus of the bronchial membrane has always been found, except when inflammation was present. The congestion of the vessels sufficiently accounts not only for the effusion of serum, but also for the tendency to pass into the inflammatory condition presented by the lungs as by other organs similarly affected. We are disposed to think, however, that this is not the whole truth, but that the interruption of innervation here, as in other cases, predisposes to inflammation, although it does not actually produce it. "The experimental history of the par vagum," remarks Dr. Reid, "furnishes an excellent illustration of the numerous difficulties with which the physiologist has to contend, from the impossibility of insulating any individual organ from its mutual actions and reactions, when he wishes to examine the order and dependence of its phenomena." Nothing, indeed, can be more various than the statements made by different experimenters of the results of their enquiries, of which an instructive summary is given by Dr. R. These differences, no doubt, arise from varieties in the conditions of the experiment; and the number of cases related in this memoir has enabled the author to point out several of these varieties, which had been unsuspected by his predecessors, whose experience was more limited. These are investigations in which no useful inference can be drawn from one or two experiments only; to avoid all sources of fallacy a large number must be made, and the points in which all agree separated from others on which there is a variation of results; and it must then be enquired to what the latter is due.

These observations apply equally to the other question which has been made by Dr. Reid a special subject of investigation—the function of the *gastric branches* of the vagus nerve—on which there has been a difference of opinion no less remarkable than on that just discussed. The experiments of Dr. Reid do not furnish grounds for positive conclusions on the subject; but they furnish important corrections of the results presented by others. He has frequently succeeded in exciting muscular contractions in the stomach by irritation of the nerve; but he has also frequently failed. It may be questioned, however, whether these movements are produced by the direct influence of the nerves, or whether they are excited by the contractions of the œsophageal filaments, which are unquestionably stimulated through the nerves. When these movements of the stomach are thus artificially induced, they commence at the cardiac orifice, and propagate themselves over a greater or less extent of the left portion of the viscus; they are evidently slower, more prolonged, and more vermicular than those of the œsophagus. These movements of the stomach, however, may take place after the section of the vagi, as is proved by some experiments presently to be mentioned, in which the food had been digested and propelled into the intestines, subsequently to this operation. "That lesion of the vagi is generally followed by vomiting (in those animals susceptible of it), loathing of food, and arrestment of the digestive process, has been incontrovertibly proved by numerous experimenters. That perfect digestion may occasionally take place after division of the vagi in the neck, even when the cut ends of the nerves are kept apart from each other, we are also fully convinced. In four of the seventeen animals experimented on for the purpose of examining the morbid changes in the lungs, we obtained sufficient evidence of the restoration of the digestive process." This evidence consisted in the acquisition of flesh and strength by the animal, which had at first suffered much from the effects of the operation; the vomiting of half-digested food, permanently reddening litmus paper; the disappearance of a considerable quantity of alimentary matter from the intestinal canal, and the existence of chyle in the lacteals. In all these cases it is to be observed that the digestive powers did not appear reestablished until the fifth day after the operation; having been in these animals, as in those which died before that period, completely arrested in the first instance. This fact accounts for the contrary results obtained by other experimenters; as it is only when circumstances favour prolonged life that such restoration can be manifested. Dr. Reid states that seven of the seventeen experiments were performed before he obtained any evidence of digestion; and that the four which furnished this followed one another almost in succession, so that it is easy to explain how some experimenters have been more successful than others when the number of their experiments is limited.

Dr. Reid next considers the effects of lesion of the vagi upon the *sensations of hunger and satiety,* and shows that these are not abolished by section of these nerves, as Brachet and others have maintained, though very probably impaired. From this and other facts he is led to the conclusion that these sensations, though referred to the stomach as their seat, originate in impressions made upon the nerves of the system at large, and have reference rather to its requirements than to the fulness or emptiness of the digestive cavities. He then adverts to the fact that the *secretion of gastric juice*, of the true acid character and solvent powers, is not always checked by section of the nerves; and he quotes the statement of Müller, that where it does appear to cease, the application of galvanism has not succeeded in reestablishing it. We hope, therefore, that physiologists will ere long be tired of quoting the oft-cited experiments of Dr. W. Philip in proof of the *dependence* of this secretion upon nervous influence, and the identity of the latter with galvanic electricity. The usual mucous secretions, too, were always found; and the inflamed appearance of the mucous membrane of the stomach described by Gendrin was never noticed. Another series of experiments was performed by Dr. Reid, with the view of testing the validity of the results obtained by Sir B. Brodie, relative to the effects of section of the nerves upon the secretions of the stomach, after the introduction of arsenious acid into the system. With this view he made five sets of comparative experiments, employing in each two dogs as nearly as possible of equal size and strength, introducing the same quantity of the poison into the system of each in the same manner, but cutting the vagi in one, and leaving them entire in the other. This *comparative* mode of experimenting is obviously the only one admissible in such an investigation. Its result was in every instance opposed to the statements of Sir B. Brodie; the quantity of the mucous and watery secretions of the stomach being nearly the same in each pair of animals subjected to experiment; so that they can no longer be referred to the influence of the eighth pair of nerves. Moreover, the appearances of inflammation were, in four out of the five cases, greatest in the animals whose vagi were left entire; and this seemed to be referrible to the longer duration of their lives after the arsenic had been introduced. The results of Sir B. Brodie's experiments may perhaps be explained by the speedy occurrence of death in the subjects of them, consequent, it may be, upon the want of sufficiently free respiration.

The following is Dr. Reid's general conclusion from his experiments on the influence of the eighth pair upon the secretive processes of the stomach, which, we think, gives a peculiarly judicious view of the nature of nervous influence upon these processes in general. "These experiments ought to be viewed in two different aspects; for while they show that the integrity of those nerves is not a condition absolutely necessary for the performance of secretion in the stomach, they yet prove that the secretions usually poured into the interior of that organ may be modified, in a most important manner, by causes acting through these nerves. We have here two perfectly separate and distinct propositions, which have sometimes not been clearly distinguished from one another. The difference between them may be illustrated in a very simple manner. The movements of a horse are independent of the rider on his back—or, in other words, the rider does not furnish the conditions necessary for the movements of the horse—but every one knows how much these movements may be influenced by the hand and heel of the rider." "When we extend our investigation into the manner in which the function of secretion is performed to the whole range of the vegetable and animal kingdoms, we can, I think, have little difficulty in arriving at the conclusion that the nerves exercise over secretion not an uniform and essential, but an occasional and controlling influence."

The *cause of death* after section of the vagi may usually, therefore, be looked for in the changes which take place in the lungs; occasionally, however, it results from inanition, produced by the derangement of the digestive organs. It is of course understood that means have been taken to ensure the access of a sufficient quantity of air into the lungs.

The following is a summary of the results obtained by Dr. Reid, as to the functions of the different portions of the pneumogastric nerve.

The *pharyngeal branches* are entirely or almost entirely motor, and move the muscles of the pharynx and soft palate.

Of the *laryngeal* branches, the superior are almost entirely sensory, supplying the mucous surface of the larynx and part of the pharynx. The few motor filaments they contain are distributed to the cricothyroid muscles. The inferior laryngeals are principally motor, and regulate the movements of the muscles attached to the arytenoid cartilages; they also furnish sensory filaments to the trachea, and a few to the larynx and pharynx. When an irritation applied to the mucous surface of the larynx produces contraction of the muscles which approximate to the arytenoid cartilages, it is by a reflex action through the spinal cord, in which the superior laryngeals probably act as the afferent nerves, and the inferior as the efferent or motor.

The *œsophageal* filaments are partly afferent and partly motor, the contact of food producing muscular contraction by reflexed stimulation. The degree in which this mode of action extends down the œsophagus seems different, however, in different species of animals.

The *cardiac* branches do not seem necessary to the sympathy between the heart's actions and mental emotions, although probably the usual channel of it.

The *pulmonary* branches seem to be the nerves chiefly concerned in transmitting to the medulla oblongata the impressions which excite respiratory movements, and are thus principally afferent nerves. It is possible that they contain motor filaments also.

The *gastric* branches appear to influence both the muscular movements of the stomach and secretions poured from its inner surface; but neither of these changes are absolutely dependent upon their constant influence.

*Edinburgh Med. and Surg. Journal. April,* 1839.

---

### *Experiments on the Blood, in connexion with the Theory of Respiration.* By John Davy, m.d. f.r.s., Assistant Inspector of Army Hospitals.

In a former Number we gave a brief notice of Dr. Davy's paper, extracted from the Proceedings of the Royal Society. Since that period the paper itself has been published in the Transactions, and we now design to give a fuller account of it, in consequence of the importance of the subjects discussed in it, in reference to the theory of animal heat.

Dr. Davy's first enquiry was to ascertain whether the blood agitated with oxygen, or with atmospheric air, is capable of absorbing either of these two gases, without entering into a state of putrefaction? The blood experimented upon was from different animals—man, the ox, sheep, dog, and cat, and in every instance was first prepared by the displacement of its fibrine, which was done by agitating it in a bottle with small pieces of sheet-lead. Whether oxygen or atmospheric air was used, there was a marked diminution in the volume of gas; but when the former was employed, the quantity absorbed was by far the greatest. Thus "sixty-two measures of arterial blood from the carotid artery, agitated with thirty-three measures of common air, produced a diminution of two measures; and sixty-three of the same arterial blood, agitated with thirteen of pure oxygen, a diminution of three; whilst sixty-three of venous blood, from the jugular vein of the same animal, agitated with thirty-three of common air, produced a diminution of six, and seventy of this blood with thirteen of oxygen a diminution of eight." It thus appeared that the blood is capable of absorbing gases, and that the quantity absorbed by venous blood is very much greater than by arterial. In every instance, both of the absorption of oxygen and of atmospheric air, the colour of the blood was changed, the venous blood became of a vermilion hue, and the colour of the arterial was rendered brighter. The experiments quoted were all made upon the blood of the same animal; but in the experiments upon the blood of different animals, the degree of absorption varied considerably, and this was more particularly the case with the blood of man. In every instance, however, absorption to a certain amount occurred. Upon this subject Dr. Davy formerly arrived at a negative conclusion; and he accounts for the discrepancy between his present and former experiments by supposing, that it has arisen chiefly from the difference of season at which the two sets of experiments were made; the first having been made during the summer at Malta, in July and August, when the temperature of the atmosphere was from eighty to ninety degrees, while the latter was made in England during the winter, in very severe weather, when the greater part of the time the temperature of the air was below the freezing point; hence he is induced to infer that the temperature of the atmosphere has a great deal to do with the capability which the blood possesses of absorbing gases, and thinks from the result of his experiments, made both at a high and low temperature, that the blood has a less power of absorbing gases at a high than at a low one. If this be the case, and if animal heat be chiefly derived from the changes which take place in the blood through the agency of respiration, as it is now very generally considered to be, and as Dr. Davy's experiments presently to be noticed seem further to show, this difference in the absorbing power of the blood at a high and low temperature may fairly be looked upon as one of the chief means of preserving that equability of bodily heat observed in most of the higher animals in winter and summer. In support of this Dr. Davy found, on carefully comparing the blood of the jugular vein and carotid artery of the sheep at Malta, during the summer, that there was no perceptible difference in their colour; in each it was less florid than the arterial blood of the same animal in England in winter, and less dark than the venous, being, as it were, intermediate between the two. Another fact in support of the same view is that he found the temperature of the right ventricle of the heart as high as 109° F. in one instance; and, in the mean of nine experiments, 107·5, whilst that of the rectum, the mean of the same number of experiments was only 104·4° F. On the question as to whether blood contains carbonic acid, which is capable of being expelled by agitation with another gas, as hydrogen or oxygen, Dr. Davy has arrived at a negative conclusion, and unfavorable to the opinion of Dr. Stevens, "at least," as Dr. D. expresses himself, "in a strict and general sense," since in one of his experiments a very small quantity was expelled, but not sufficient to warrant the conclusion that this is usually the case when blood charged with carbonic acid gas is agitated with either of the other two gases. With respect to the condition of the alkali in the blood as regards carbonic acid, the experiments all led to the conclusion, that it is in the state of sesqui-carbonate. On the question, whether blood contains any air capable of being extracted by the air-pump, Dr. Davy again arrives at a conclusion different from that at which he arrived on the same subject in 1818. The presence of pure gas in the blood, extractable by the air-pump, was stated at that time by Sir Everard Home upon the authority of Mr. Brande, but the fact was not then confirmed; on the contrary, many celebrated physiologists and chemists arrived at a different conclusion, until the experiments of MM. Bischoff and Magnus. Mr. Brande had found that the quantity of gas amounted to as much as two cubic inches from one ounce of blood. This enormous quantity was so remarkable, that it was suspected there must have been some mistake; but the recent experiments of MM. Bischoff and Magnus, confirmed by Dr. Davy, render it most probable that Mr. Brande was correct, and that the negative results formerly obtained by Dr. Davy and others arose from the exhaustion of the air in the receiver not being carried sufficiently far. Indeed, Dr. Davy's present experiments almost prove this to have been the case, since it is only when the exhaustion is nearly or quite complete that the gas is evolved, and the evolution then takes place so rapidly and continuously, that the fact is indisputable. In all the experiments the blood operated upon has been obtained from animals without being allowed to come at all in contact with atmospheric air. In one set of experiments the jugular vein or carotid artery was exposed, two ligatures placed upon it, and then cut through, and the cut end of one connected with a phial filled with boiled distilled water, and the blood then allowed to flow until the water was believed to have been all dispersed; in another experiment, the blood was allowed to coagulate in the jugular vein between two ligatures, and the results in this instance were extremely interesting. At first there was no more air evolved under the air-pump than might fairly have been supposed to have adhered to the stopper of the bottle in which the coagulum was inclosed, after which there was no evolution for about five minutes, which thus led to the opinion that no gas was contained in the blood. At the expiration of that time, the exhaustion in the receiver being nearly complete, a film or bubble burst with considerable violence, and a bubbling commenced which was continued with some activity, thus rendering the fact of the extrication of gas indisputable. The quantity of gas extricated in the different experiments was exceedingly variable, so that Dr. Davy concludes that the quantity contained in the blood of different individuals, and in different states of health, is subject to much variation. No disengagement of air from the blood could be obtained while the blood was confined in a portion of the vessel between ligatures, so that a very slight compression appears to be sufficient to confine the air in the blood, or prevent it from assuming the elastic state. Dr. Davy differs from Magnus in his opinion respecting the composition of the gas extricated from the blood by means of the air-pump, in believing it to be entirely carbonic acid, and that there is neither oxygen nor azote, and this opinion he founds upon the fact that when potassa was mixed with the blood, no gas whatever was extricated, the whole being, as he supposes, absorbed by the potassa, which is not capable of absorbing either oxygen or azote. But on the question as to whether oxygen is contained in the blood, but which cannot be extricated by means of the air-pump, Dr. Davy was led to the affirmative. Both oxygen gas and nitrous gas are capable of combining with the blood, but cannot afterwards be separated from it again by means of the air-pump. From the circumstance that Dr. Davy had not employed so complete an apparatus in his experiments as was employed by Magnus, it is reasonable to expect that his results might not be so conclusive, and that the attraction of oxygen for the blood is greater than that of carbonic acid; consequently, that it is liberated with greater difficulty, and perhaps not until exhaustion in the receiver is complete; and this may be the case also with regard to azote. The important question therefore yet remains, does the blood contain oxygen or azote in a free state? If it be denied that azote enters into combination with, or is contained in a free state in the blood, from whence is derived the azote which constitutes so large a proportion of the primary constituents of animal tissues, particularly in those animals which subsist entirely upon food which contains not a particle of azote?—the graminivorous feeders—sheep, oxen, &c. This is a question often started, but still worthy of further enquiry. Another important fact examined by Dr. Davy is, whether there is any evolution of heat from blood when agitated with oxygen. Dr. Davy has distinctly proved this to be the case, care having been taken to ensure a correct result.

On the whole we think this paper of Dr. Davy's one that is deserving of considerable notice, as tending to throw much additional light upon a very difficult and most important subject. *Phil. Trans.* Part II. 1838.

---

### *Observations on the Blood after Death.* By John Davy, M.D. F.R.S., Assistant-Inspector of Army Hospitals.

This is an important communication, of which we can only give an outline. The following table notices the state of the blood in persons dying of various diseases on the island of Malta, from 1828 to 1835.

| | |
|---|---|
| Total number of fatal cases examined | 249 |
| Heart collapsed, containing either no blood, or very minute quantities | 6 |
| Blood liquid, and not coagulating on exposure to the air | 9 |
| Partly liquid, partly coagulated, former not coagulating on exposure | 3 |
| Partly liquid, partly coagulated, former coagulating on exposure | 6 |
| Liquid and coagulating on exposure | 4 |
| Coagulated and containing fibrinous concretions | 105 |
| Coagulated without fibrinous concretions, the coagulum soft | 10 |
| Grumous, not distinctly coagulated, without fibrinous concretions | 2 |
| Liquid and frothy, without any fibrinous concretions | 1 |
| Blood florid | 1 |
| Fibrinous concretions without any cruor. | 1 |
| Blood coagulated in both ventricles and broken up | 5 |
| Coagulated and broken up in left ventricle, not in right | 11 |
| Coagulated in right ventricle, and broken up; left ventricle empty | 1 |
| Cases in which the state of the blood was not noticed | 85 |

After some interesting remarks on these appearances, a second table is given of thirty-five cases, recently observed at Fort Pitt, in which attention was paid, not only to the state of the blood in relation to coagulation, but also to its state in relation to carbonic acid gas. The method employed was briefly the following. In every instance, after the removal of any fluid that might be found in the pericardium, the great vessels within it were divided; the blood which flowed out was collected and put into a bottle and secured with a glass-stopper, for subsequent observation and experiment. The cavities of the heart and their contents were next examined. Sometimes, the blood set apart, was seen again the same day, to ascertain, whether it had coagulated, or remained liquid; but more frequently, not until the following morning, when also commonly it was subjected to agitation in atmospheric air, included in a double-mouthed bottle, provided with stop-cocks, to one of which was attached a bent tube, for communication with a pneumatic trough.

The following are some of Dr. Davy's remarks on these appearances.

The instances in the first table were strongly indicative of the variable quality of the blood after death from disease; and the particular instances just given are as strongly corroborative of the same. The variable quality, as far as appreciable by the senses, seems to depend on the lymph, which, as regards coagulability, has appeared wonderfully little constant; in some instances, retaining its power of coagulation more than twenty-six hours after death; in others, coagulating almost immediately after the fatal event; and in some previous to that event. In a large number of cases, the lymph appears to have existed in the blood, possessed of the quality in question in different degrees. This is clearly to be inferred from the results of the *post mortem* inspections; namely, from finding at the same time, and in the same body, in the cavities of the heart, liquid coagulable cruor, soft crassamentum, and firm fibrinous concretions.

The fibrinous concretions, which are so frequently found in the heart after death, are far from being devoid of interest. They, too, are very various, both as regards form and consistence. They are also very various in relation to the manner in which they are attached to the heart. I have mentioned one instance, in which serum was collected in the interior of a mass; the inner part was soft, the outer surface firm. Some masses seem to have a certain regularity of structure. Some have the appearance of being inflamed, being blood-shot from entangled red particles of blood. Some exhibit the appearance of abscess, and contain, as it were in a sac, a puriform matter, generated by a peculiar process of softening. Generally, they tend to give the idea of the operation of an organizing principle, a *nisus formativus*, more or less active, until putrefaction commence, by which the animal matter is converted into common matter.

The different varieties of fibrinous concretions are admirably illustrative of certain morbid appearances, especially false membranes, bands of adhesion, the state of the cellular tissue in different kinds of œdema and induration; and, I may add, hepatization of the lung, with and without softening; and a similar state of the spleen, and perhaps tubercles and their softening. When I have examined, microscopically, the matter yielded by the lung which has been hepatized, undergoing softening, I have found it very similar to the matter into which a fibrinous concretion is converted, whether in the ventricles of the heart or in a vein, composed of particles of different sizes, most of them approaching the spherical in form, and some of them very like pus globules; and, I have found the same kind of matter in softened spleen, and also in softened tubercular matter.

No subject, probably, in pathology, is more deserving of careful investigation than lymph, in connexion with its varieties, supposing either that there are many different species of it, or that it is liable to sudden and great changes of quality. Relative to the former, take for instance the phlebolite, in its different stages of induration, from soft lymph becoming of cartilaginous firmness and appearance; the fibrinous layers deposited in an aneurismal sac, resisting changes, occasionally for years; and the fibrinous concretions which are formed in the large veins, especially in phthisis, which so soon soften and in part almost liquefy; how great is the diversity, how difficult in the present state of our knowledge the explanation.

Nor is the liquid cruor or the blood remaining either in part or altogether liquid after death, whether for a definite or indefinite time, without interest and importance, especially as regards pathological research, and the distinguishing between the effects of disease and *post mortem* effects, in the inspection of bodies. So long as the blood is liquid after death, it must necessarily observe the laws to which fluids are subject under similar circumstances; will accumulate in the dependent parts and where there is least resistance; and, should there be considerable pressure exerted, as from the disengagement of air in the stomach and intestines, it will appear as if injected in the organs removed from such pressure, whether included within unyielding parietes, as the brain, subject to occupy less volume from greater reduction of temperature after death than its hard, and, during life, cooler case; or contained in yielding envelopes, as the lungs in the pleuræ and other parts of the body generally, in the common integuments and in their peculiar capsules. In a considerable number of examples given in the table, cruor was found in the heart more than twenty-four hours after death. This cruor, in producing false appearances of inflammation and congestion, would act much in the same manner as liquid blood; and, in estimating the phenomena which are presented on dissection, much the same allowance should be made for it, or serious errors will be unavoidable.

From the experiments given in the table on the agitation in air of the blood collected after death, it would appear, that in the greater number of instances, gas was disengaged. In several instances, the air thus liberated was tried, and was found to be carbonic acid gas; proving that there was an excess of this acid in the blood. This excess, I am disposed to infer, especially from the results of experiments which I have recently made on healthy blood, an account of which is published in the Philosophical Transactions for the present year, is the effect of disease, and is particularly apt to take place in the act of dying, when the powers of secretion seem to be arrested, and when carbonic acid probably ceases to be eliminated in the lungs. In accordance with this, with very few exceptions, is the uniform dark colour of the blood usually observed after death, and equally in the left and in the right ventricle of the heart; and also, the little suffering commonly witnessed in dying, diminished sensation probably as in birth, or stupor, or coma, usually preceding and ushering in the fatal event.

From the results referred to, and from a few which I have obtained in operating on blood taken from persons labouring under disease, I am disposed to think that carbonic acid acts a very important part in the economy of life; and is connected when in excess, if not with the production of particular diseases, at least with their modification and progress and the production of certain symptoms, and that the careful examination of the blood, in relation to this acid, is an enquiry much wanted, and is likely to reward richly those who engage in it. At present, there is a great difference of opinion derived from experiment, respecting the carbonic acid in the blood; some enquirers, as Dr. Stevens, to whom belongs the merit of having opened this path of research, considering it, in the healthiest condition, always in excess and capable of being liberated from venous blood and even from arterial, by the physical agencies in play in respiration; whilst others take an opposite view, and deny that any free acid exists in the blood in its healthy normal state, or can be separated from it excepting by processes in which it is formed from its elements. This discrepancy, the enquiry I have mentioned, will probably explain or reconcile. It will elucidate also, probably, some other obscure points, which might be mentioned, especially were it extended to other fluids, as the urine and bile. I may mention, incidentally, that whenever I have obtained indications of the disengagement of carbonic acid from the blood after death, they have been afforded by the fluids spoken of, in a very limited number of trials, and also by any effused serous fluid collected at the same time, and subjected to the same test.

*Edinburgh Med. and Surg. Journal. April,* 1839.

